# Effect of smartphone-based stress management programs on depression and anxiety of hospital nurses in Vietnam: a three-arm randomized controlled trial

**DOI:** 10.1038/s41598-021-90320-5

**Published:** 2021-05-31

**Authors:** Kotaro Imamura, Thuy Thi Thu Tran, Huong Thanh Nguyen, Natsu Sasaki, Kazuto Kuribayashi, Asuka Sakuraya, Thu Minh Bui, Anh Quoc Nguyen, Quynh Thuy Nguyen, Nga Thi Nguyen, Kien Trung Nguyen, Giang Thi Huong Nguyen, Xuyen Thi Ngoc Tran, Tien Quang Truong, Melvyn Weibin Zhang, Harry Minas, Yuki Sekiya, Kazuhiro Watanabe, Akizumi Tsutsumi, Norito Kawakami

**Affiliations:** 1 Department of Mental Health, Graduate School of Medicine, The University of Tokyo, 7-3-1, Hongo, Bunkyo-ku, Tokyo, 113-0033 Japan; 2 Department of Occupational Health and Safety, Faculty of Environmental and Occupational Health, Hanoi University of Public Health, Hanoi, Vietnam; 3 Faculty of Social and Behavioral Sciences, Hanoi University of Public Health, Hanoi, Vietnam; 4 Department of Mental Health and Psychiatric Nursing, Graduate School of Health Care Sciences, Tokyo Medical and Dental University, Tokyo, Japan; 5 Division of Public Health, Department of Hygiene and Public Health, School of Medicine, Tokyo Women’s Medical University, Tokyo, Japan; 6 Nursing Office, Bach Mai Hospital, Hanoi, Vietnam; 7 Bach Mai hospital, Hanoi, Vietnam; 8 National Addiction Management Service, Institute of Mental Health, Singapore, Singapore; 9 Melbourne School of Population and Global Health, The University of Melbourne, Melbourne, Australia; 10 Department of Public Health, Kitasato University School of Medicine, Sagamihara, Japan

**Keywords:** Medical research, Clinical trial design, Randomized controlled trials, Health care, Occupational health, Diseases, Psychiatric disorders, Anxiety, Depression

## Abstract

There are growing concerns on stress among nurses in low- and middle-income countries (LMICs) in South-East Asia. It is important to improve mental health among nurses in these countries. The objective of this study was to examine the efficacy of two types of newly developed smartphone-based stress management programs in improving depressive and anxiety symptoms among hospital nurses in Vietnam. This study was a three-arm (including two intervention groups and one control group) randomized trial. Participants were recruited from nurses in a large general hospital in Hanoi, Vietnam. Two types (free-choice and fixed sequential order) of smartphone-based stress management programs were developed. Participants were randomly allocated to Program A (a free-choice, multimodule stress management), Program B (a fixed-order, internet cognitive behavioral therapy, iCBT), or a control group (treatment as usual). The depressive and anxiety symptoms were measured by using the Depression Anxiety and Stress Scales at baseline, 3-, and 7-month follow-up surveys. 951 participants were randomly allocated to each of the three groups. Program B showed a statistically significant effect on improving depressive symptoms at 3-month (p = 0.048), but not at 7-month (p = 0.92); Cohen’s d was − 0.18 (95% CI − 0.34 to − 0.02) and 0.03 (95% CI − 1.00 to 1.05), respectively. Program A failed to show a significant intervention effect on any of the outcomes at 3- or 7-month follow-up (p > 0.05). Despite the small effect size, the present fixed-order iCBT program seems effective in improving depression of hospital nurses in Vietnam. A public health impact of the intervention can be scalable, when considering its accessibility and minimal cost.

Trial registration number: The study protocol is registered at the UMIN Clinical Trials Registry (UMINCTR; ID = UMIN000033139). Registered date of the protocol is 1st Jul. 2018. https://upload.umin.ac.jp/cgi-open-bin/ctr_e/ctr_view.cgi?recptno=R000037796

## Introduction

Population in many low- and middle-income countries (LMICs) will age rapidly by 2050^1^, as they have already in most high-income countries. With the population aging, the increased medical needs have caused a severe deficit in the nursing workforce, especially in South-East Asia including LMICs^2^. A shortage of health service providers such as nurses is already reported in many LMICs in South-East Asia^2,3^. In addition to increasing numbers of people requiring care, the growing demands from service users (i.e., patients and families) for improved quality of care have put pressure on and have caused distress among nurses^4–6^. Also, in the recent pandemic of new coronavirus disease (COVID-19), nurses experienced extreme stress in caring for patients infected under the risk of their own infection^7^.

In LMICs in South-East Asia, including in Vietnam, healthcare professionals including hospital nurses have reported high stress^8^. A recent study reported that more than 45% of hospital nurses in Vietnam had any of depression, anxiety, and stress symptoms^8^. It is known that psychological distress (i.e., depression and anxiety) among workers results in substantial deterioration in workers’ quality of life and economic loss in the workplace, and in decreased work performance, especially quality of care provided by nurses^9–13^. There are growing concerns for mental health problems among nurses and other healthcare professionals in LMICs in South-East Asia^14^. It is important to manage work-related stress and to improve mental health among nurses to ensure health and well-being of nurses and the quality of care they provide.

Especially in low-resource settings in LMICs, there is a substantial need for accessible and low-cost intervention programs. Cognitive behavioral therapy (CBT) has been shown to be effective for reducing depression and anxiety among workers^15,16^, including healthcare workers. In a healthcare setting, an individual-oriented approach such as CBT has been reported to be more effective than an organizational approach^17^. To deliver stress management based on CBT, a computerized CBT delivered via the internet (iCBT) has been shown to be an effective, low-cost and highly accessible approach^18–20^. One randomized controlled trial (RCT) reported that a web-based stress management program including cognitive behavioral techniques reduced perceived work-related stress among nurses in the USA^21^, while no significant effect was found on emotional or physical symptoms. There has been no RCT that has investigated effectiveness of this approach among nurses in LMICs. Recently, the smartphone penetration rate in LMCIs has rapidly increased: for instance, in Vietnam, the rate of people using smartphones was 68–84% from rural to urban area in 2017^22^. In the “new normal” era during and after the outbreak of the COVID-19, it is strongly recommended to switch the mode of delivery of a stress management training from a face-to-face class-based program to an online program^7^. It is important and urgent to clarify the effectiveness of an iCBT program for improving depression and anxiety among nurses in LMICs, such as Vietnam.

A major challenge in implementation of iCBT interventions is low adherence. A systematic review reported a completion rate of iCBT programs in the workplace was approximately 40%^18^. A useful strategy to promote adherence in web-based interventions is incorporating tailoring^23^. An approach that may increase completion rates and effectiveness of iCBT programs is to allow participants to choose modules based on their preference. It has been reported that a free-choice program in European countries had a higher completion rate and was more effective than a fixed order program^24^. However, it is not clear whether these findings are applicable to countries in other cultures, such as those in South-East Asia. In addition to test the effectiveness of an iCBT program for improving depression and anxiety among nurses, it would be worthwhile to investigate which of free-choice or fixed order iCBT program could achieve better completion rate in Asian countries that have collectivism- and hierarchy-oriented organizational cultures^25^.

Depending on a target population of an intervention, there are different approaches: (1) universal prevention that targets the whole population including those with and without symptoms; and (2) selective and indicated interventions that target high-risk or symptomatic groups^26^. While the latter approach often yielded greater effect size for mental health outcomes than the universal prevention in web-based psychological interventions in the workplace^18^, the universal prevention has a merit because it does not only improve symptoms in a symptomatic group, but also prevent the onset of symptoms among an asymptomatic group^27^. The universal prevention for mental health also has an advantage in its less stigmatizing nature^28^. Thus, it has been recognized as a promising approach in the workplace^29^. It is crucial to test the effectiveness of an iCBT program as the universal prevention among nurses in LMICs.

### Objective

The objective of this RCT was to examine the universal prevention effect of two types of newly developed smartphone-based iCBT stress management programs (i.e., free-choice sequence and fixed sequential order) compared with a control group in improving depressive and anxiety symptoms as primary outcomes at 3-month and 7-month follow-ups among hospital nurses in Vietnam, using a three-arm trial design.

## Methods

### Trial design

This study was a three-arm (including two different intervention groups), parallel-group, treatment as usual (TAU)-controlled, non-blinded randomized trial. The allocation ratio of the intervention groups to the control group was 1:1:1. Follow-up surveys were conducted at 3- and 7-month after the baseline survey. The study procedures have been approved by the Research Ethics Review Board of Graduate School of Medicine/Faculty of Medicine, the University of Tokyo (no 11991) and the Ethical Review Board for Biomedical Research of Hanoi University of Public Health (no 346/2018/YTCC-HD3). The study protocol was registered at the University Hospital Medical Information Network Clinical Trials Registry (ID = UMIN000033139). All methods were performed in accordance with the approved study plan, as well as with relevant guidelines and regulations. Registered date of the protocol is 1st Jul. 2018. The protocol article for this trial is available^30^. This manuscript conforms to the Consolidated Standards of Reporting Trials (CONSORT) guidelines^31^.

### Participants

All participants were recruited from a large general hospital in Hanoi, Vietnam. Participants received from the head nurse of each department in the hospital a self-administered questionnaire, return envelope, documents explaining the purpose of the study, the privacy policy, and an informed consent form. A clinical research coordinator (CRC; TTTT) provided a briefing session for participants to fully explain the study objectives and procedures. Candidates were informed that their participation was voluntary, that even after voluntarily participating they could withdraw from the study at any time without stating the reason and that neither participation nor withdrawal would cause any advantage or disadvantage to them. After the explanation, written informed consent was obtained from all participants. Participants were given a small monetary reward (7 USD) for completing the baseline and follow-up questionnaires. The inclusion criteria at the baseline survey were as follows: (1) Currently employed full time as a registered nurse, and (2) Can access the internet via a mobile device such as a smartphone. The exclusion criteria were as follows: (1) Plan to change or quit the job in the next 7 months, (2) Assistant nurses and helpers, (3) Non-regular or part-time employed, (4) Sick leave for 15 or more days for a physical or mental condition in the past 3 months, and (5) Current treatment for a mental health problem from a mental health professional. However, in this study, exclusion criteria nos. 4 and 5 were withdrawn before the start of the baseline survey (see details in “Changes to the protocol” in the Methods).

### Intervention programs

Two smartphone-based six-module stress management programs were used. One (Program A) was a free-choice, multimodule stress management program in which respondents were allowed to select one module per week in any order they preferred. The other (Program B) was a fixed-sequence, multimodule stress management program in which respondents were required to study modules in a fixed order, one module per week. For both programs, it took about 15 minutes to complete each module. Participants in the intervention groups were invited to access and study the allocated program by using the app downloaded to their smartphones, while they were also able to access to the program from PCs or other devises. To develop these programs considering the working situation and work culture of nurses in Vietnam, 30 senior nurses of the target hospital (who were not participants in the study) were invited to a meeting with researchers to review and comment on the intervention programs based on their priorities, experience, and preferences. Full details of these programs may be found in the published study protocol paper^30^. Briefly, Program A included behavioral activation (module 1), cognitive restructuring (module 2), problem-solving (module 3), assertiveness (module 4), self-compassion (module 5), and job crafting (module 6). Participants chose one module per week in any order they preferred. Program B also included six modules that provide CBT-based stress management skills, including a transactional model of stress and coping (module 1), self-case formulation based on cognitive behavioral model (module 2), behavioral activation (module 3), cognitive restructuring (module 4), cognitive restructuring and relaxation (module 5), and problem-solving (module 6). The six modules were presented in a fixed order, with one module accessible per week, from module 1 to module 6. Major features of the programs compared to other smartphone apps for mental health^32,33^ are as follow: (1) Accessibility: These programs were accessible using smartphone apps downloadable from Apple App Store and Google Play, as well as from PCs and other devices connected to the Internet. (2) Program content: Both programs were developed based on previous evidence-based web-based stress management programs that showed the universal prevention effect on psychological distress or depression of office workers in Japan^34,35^. The program content was developed to primarily target nurses with none or mild symptoms of stress. (3) Adaptation to the target population: the programs were developed and modified with an extensive discussion with a group of senior nurses in Vietnam so that the content was relevant to and appropriate for the working situation and work culture of nurses in Vietnam. For instance, case stories used to illustrate the stress process and stress management were newly developed, considering common stressors in the target population suggested in the discussion with the senior nurses, such as job overload, role conflict, and work-family interference. (4) Other functions: The program provided a function for self-assessment of participants’ mood by using the Kessler 6 scale of psychological distress^36,37^ to motivate the participants to study the programs. No track system of behavioral or physiological parameters such as sleep or exercise was included. (5) Design and visual appeals: The program used cartoon characters^35^ who talked on the study topic and guided participants through the learning. The cartoon and other images were developed acceptable to the hospital settings and cultures in Vietnam. (6) Learning support: The programs were fully-automated and self-guided, provided without any professional or therapist support during the learning, to minimize the running cost. No primary task support, dialogue support, or social support was provided.

### Intervention groups

Participants in the intervention groups were required to complete Program A or B within 10 weeks after the baseline survey. Participants were provided with their own ID and password to sign in to the program. Before the start of the program, participants in the intervention groups were given a workshop on how to download the apps and to read an introduction module, which was identical for the two programs and provided a general overview and how to use the programs. They were asked not to disclose this information learned from the programs to others nurses. If participants did not complete any module on time, they were reminded by an instant message from the CRC. In addition, informal group chat (via Viber, Zalo, Facebook messenger) and hotline 24/7 by the research team facilitated the intervention groups to use the programs. The nursing department of the hospital and senior managers who did not participate in the study also sent message to the whole group to encourage the intervention groups to use the programs, although they were blinded to the group allocation. These technical support and reminders did not include professional support for studying CBT.

### Control group

Participants in the control group did not receive any intervention program during the intervention and follow-up periods (7 months). Participants in both the intervention group and the control group were able to use an internal employee assistance program service of the hospital as a TAU. Participants in the control group were provided the opportunity to use the intervention programs after the 7-month follow-up.

### Outcome measures

All outcomes were assessed at baseline, 3-month, and 7-month follow-ups, using a paper-based self-administrated survey questionnaire. The questionnaires were distributed by an independent research assistant who was blinded to the group allocation and returned by participants in sealed envelopes.

### Primary outcomes: *Depression and anxiety*

The primary outcomes measured in this RCT were depressive and anxiety symptoms. To assess these outcomes, the depression and anxiety subscales of the short 21-item version of the Depression Anxiety and Stress Scales (DASS 21, seven items in each subscale) were used^38^. The DASS is a widely used screening tool to assess symptoms of depression, anxiety, and stress experienced in the previous 7 days in community settings^39^. The depression subscale measures dysphoria, hopelessness, and devaluation of life, among others. The anxiety subscale measures autonomic arousal, skeletal musculature symptoms, and situational anxiety, among others. Items were scored on a 4-point scale ranging from 0 (*did not apply to me at all*) to 3 (*applied to me very much, or most of the time*). In order to yield equivalent scores to the full version of DASS (42-item), the total score of each scale was multiplied by 2 with ranges from 0 to 42^38,39^. The ranges of cut-off scores of the depression subscale (DASS21-D) are as follows; normal (0 to 9), mild (10 to 13), moderate (14 to 20), severe (21 to 27), and extremely severe (28 or more). The ranges of cut-off scores of the anxiety subscale (DASS21-A) are as follows; normal (0 to 7), mild (8 to 9), moderate (10 to 14), severe (15 to 19), and extremely severe (20 or more). A Vietnamese version of DASS 21 has been developed and tested, and its internal consistency (Cronbach’s alpha) of each DASS21-D and DASS21-A subscale was acceptable (0.72 and 0.77, respectively) and validity has been confirmed^40^. According to that study, DASS21-D predicts depression with a cut-off score 10 or more, with good screening performance (sensitivity = 0.81, specificity = 0.77, and area under the curve = 0.80)^40^.

### Process evaluation

#### Program satisfaction, usefulness, and usage

Participants in the intervention groups were asked to rate their satisfaction with the intervention program at 3-month follow-up survey using one item with five response options (*very satisfied* to *very dissatisfied*). In addition, participants in the intervention groups were asked to rate the usefulness of the intervention program at 3- and 7-month follow-up surveys using one item with five response options (*very useful* to very *useless*). To evaluate a difference in adherence to the two intervention programs, the usage rates (i.e., completion rates) of the intervention programs were collected from the records of the apps system.

#### Assessment of the adverse event or side effect of the programs

To assess the occurrence of any adverse event or side effect due to the use of the intervention programs, participants in the intervention groups were asked the following question at 7-month follow-up survey: “Did you experience any side effect or bad experiences due to the use of the program?,” with a response option as follows; “no,” “took much time,” “interfered daily work,” “interfered personal life,” “had any mental problem,” “had any physical problem,” and “others.” This scale was originally developed.

#### Contamination of information

To evaluate the degree of contamination of information from the intervention groups to the control group, the control group participants were asked the following question at 3- and 7-month follow-up surveys: “During the past 3 (or 7) months (or any time after the baseline survey to the point of follow-up surveys), have you got to know information on stress management from your colleagues who used any smartphone-based stress management programs?,” with a response option yes/no. This scale was originally developed.

#### Demographic characteristics

Demographic data, such as age, gender, marital status, education, service years, and overtime hours during the past month were collected at baseline survey. Information on whether the participants had been diagnosed with any chronic diseases before the baseline survey was collected in the 3-month follow-up survey.

#### Sample size calculation

A required sample size was calculated for the depressive symptoms assessed by DASS 21. Previous meta-analyses of web-based psychological intervention on improving workers' mental health in the workplace yielded effect sizes of 0.23 to 0.37^18,19^. To detect a small effect size (i.e., 0.25) or more at an alpha error rate of 0.05 and a beta error rate of 0.15, the estimated sample size was 289 participants in each group. The statistical power was calculated using the G*Power 3.1 program^41^.

#### Randomization

Participants who fulfilled the eligibility criteria were randomly allocated to one of the three trial arms (two intervention groups or control group). Stratified permuted-block randomization was conducted. The block sizes of this study were fixed to 3. Participants were stratified into two strata according to the depression subscale score of DASS 21 (≥ 10 or < 10) in the baseline survey^40^. A stratified permuted block random table was generated by an independent biostatistician. Enrollment was conducted by the CRC, and assignment was conducted by an independent research assistant. The stratified permuted-block random table was password protected and blinded to the researcher. Only the research assistant could access it during the work of random allocation.

### Statistical analyses

For the main pooled analysis, a mixed model for repeated measures (MMRM) conditional growth model analysis with an unstructured covariance matrix was conducted using a group (intervention and control) × time (baseline, 3-month and 7-month follow-ups) interaction as an indicator of intervention effect. For sensitivity analysis, a similar MMRM, but using the analysis of variance model, with an unstructured covariance matrix was conducted. In each analysis, the intervention effects for Programs A and B were simultaneously tested in a same model. A stratification factor that was used to stratify the participants into two strata (i.e., ≥ 10 or < 10 of DASS depression subscale score at the baseline survey) before the randomization was always included in the model^42^.

At the baseline survey, if the number of missing items was less than half of the number of total items, the missing values were imputed applying values calculated according to the following equation: the mean value, timed the number of total items, divided by the number of missing items. Otherwise, if the number of missing items were more than half of the number of total items, the case was not imputed and treated as the missing. Missing values at follow-up surveys were imputed applying the maximum likelihood estimation using the MIXED procedure. An intention-to-treat principle was applied.

The effect size was estimated in two ways. First, we estimated a regression coefficient for a group (each of the two intervention groups vs. the control group) x time (baseline and two follow-ups) interaction using the MIXED procedure, which was converted to an effect size by dividing by a pooled SD at baseline and at follow-ups. Second, we calculated Cohen’s d among participants who completed surveys at baseline and each follow-up. The level of statistical significance for all analyses in this study were set at 0.05 (two-tailed), and 95% CIs were calculated. For process measures, the χ^2^ test was performed to examine the difference between the two intervention groups. All statistical analyses were conducted using the SPSS Statistics V.22.0 (IBM).

### Subgroup analysis

Subgroup analyses were conducted separately among respondents using the stratification factor (i.e., participants who scored ≥ 10 in DASS depression subscale at the baseline survey) according to a priori-defined subgroups (selective intervention effect).

### Changes to the protocol

One major change made to the protocol was in the eligibility criteria. Originally, five exclusion criteria were set (see above). Before the start of this study, criteria nos. 4 and 5 were canceled due to the anticipation of a low participation rate and to minimize restrictions applied to select participants in order to know the effect of the programs in the real setting as a pragmatic trial^43^.

## Results

### Recruitment

**Figure 1:**
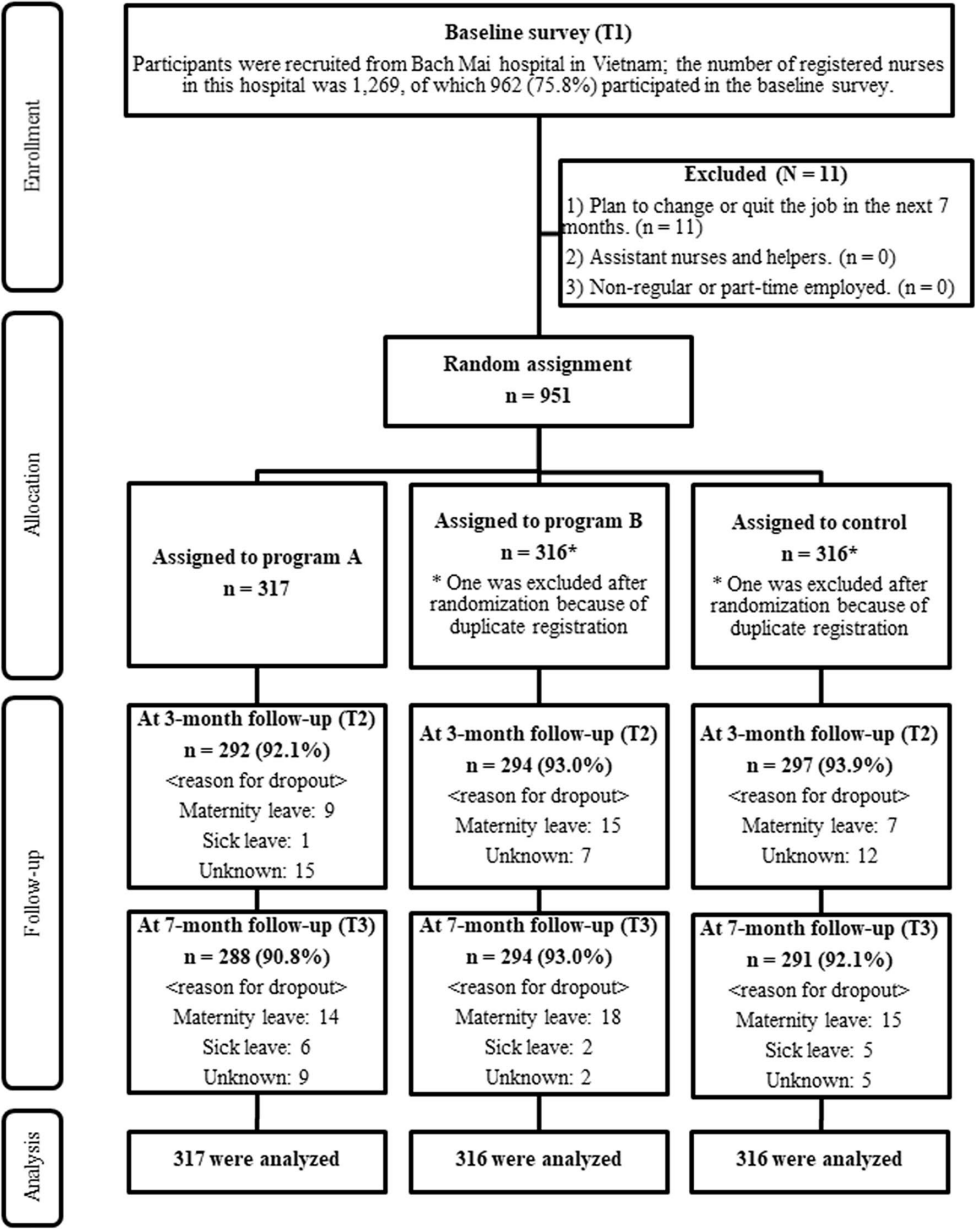
Flow diagram of the progress through the phases of a three-group randomized controlled trial of the effect of smartphone-based stress management programs on reducing depression and anxiety among hospital nurses in Vietnam.

Recruitment and the baseline survey were conducted in September 2018. The intervention and control groups were assessed at approximately 3 months (January 2019) and 7 months (May 2019) after the baseline survey. The participant flowchart is shown in Fig. 1. In total, 75.8% of workers (962/1,269) participated in the baseline survey. Out of those workers, 11 were excluded based on exclusion criterion 1. 951 met the eligibility criteria of this study and were randomly allocated to each of the three groups (two intervention and one control groups) with 317 in each group. After the random assignment, one participant in the intervention group (Program B) and one in the control group were excluded because of duplicate registration.

### Baseline characteristics

Demographic characteristics are presented in Table 1. In the whole sample, most participants were females, married, received vocational school or university education, and did not report having chronic diseases. Among the three groups, demographic characteristics of participants were similar. About 24% of participants in each of the three groups had mild depressive symptoms (i.e., scored 10 or more on DASS depression subscale).

**Table 1:** Baseline characteristics of participants in the intervention and control groups.

	Program A (n = 317)	Program B (n = 316)	Control (n = 316)
Variables	n (%)/mean (SD)	n (%)/mean (SD)	n (%)/mean (SD)
Age (years)	33.7 (7.3)	32.8 (6.6)	32.8 (6.4)
**Gender**			
Male	39 (12.3)	56 (17.7)	48 (15.2)
Female	278 (87.7)	260 (82.3)	268 (84.8)
**Marital status**			
Never married	40 (12.6)	44 (13.9)	53 (16.8)
Married	270 (85.2)	267 (84.5)	256 (81.0)
Divorced	5 (1.6)	5 (1.6)	6 (1.9)
Unknown	2 (0.6)	0 (0)	1 (0.3)
**Education**			
Vocation school	151 (47.6)	142 (44.9)	150 (47.5)
College	39 (12.3)	50 (15.8)	48 (15.2)
University	118 (37.2)	119 (37.7)	111 (35.1)
Graduate school	6 (1.9)	5 (1.6)	6 (1.9)
Unknown	3 (0.9)	0 (0)	1 (0.3)
Service years	10.2 (7.5)	8.9 (6.8)	9.0 (6.5)
Overtime work hours past a month	15.1 (28.8)	19.0 (25.9)	19.3 (30.4)
At least one chronic disease before baseline survey*	93 (31.8)	73 (24.8)	84 (28.3)
10 or more on DASS depression subscale score	77 (24.3)	77 (24.4)	77 (24.4)

*This variable was collected at 3-month follow-up survey.

### Effect of the intervention programs on depressive and anxiety symptoms

The average scores of DASS depression and anxiety subscales decreased from baseline to 3-month follow-up, but increased again at the 7-month follow-up in the two intervention groups (Table 2). Program B showed a significant effect size in reducing depression score at 3-month follow-up compared to the control group (d = − 0.18 [95% CI − 0.34 to − 0.02]). The MMRM analyses revealed that Program B showed a significant estimated effect on depression subscale score at 3-month follow-up (t = − 1.98, p = 0.048), but not at 7-month follow-up (t = 0.10, p = 0.92) (Table 3). Program B did not show a significant estimated effect on anxiety subscale score at 3- or 7-month follow-up. Program A did not show a significant estimated effect on depression or anxiety subscale score at 3- or 7-month follow-up.

**Table 2:** Means (SDs) of outcome variables at baseline, 3- and 7-month follow-up in the intervention and control groups.

DASS subscales	Program A (free-choice)		Program B (fixed-sequence)		Control		Effect size (program A vs control)	Effect size (Program B vs control)
	n	Mean (SD)	n	Mean (SD)	n	Mean (SD)	Cohen’s d (95% CI)	Cohen’s d (95% CI)
**Depression**								
Baseline	317	5.8 (5.8)	316	6.1 (5.6)	316	6.3 (6.2)	–	−
3 M follow-up	291	3.5 (5.0)	293	3.4 (4.3)	296	4.5 (5.4)	− 0.08 (− 0.24 to 0.08)	− 0.18 (− 0.34 to − 0.02)
7 M follow-up	288	4.5 (5.2)	294	4.6 (5.5)	288	4.7 (6.3)	0.07 (− 0.10 to 0.23)	0.01 (− 0.15 to 0.17)
**Anxiety**								
Baseline	317	7.2 (5.8)	316	8.0 (6.4)	316	8.1 (6.5)	−	−
3 M follow-up	291	4.9 (5.3)	293	5.0 (5.2)	296	5.7 (5.9)	0.00002 (− 0.16 to 0.16)	− 0.14 (− 0.30 to 0.02)
7 M follow-up	288	5.3 (5.0)	294	5.9 (6.0)	288	6.0 (6.3)	0.03 (− 0.13 to 0.20)	− 0.01 (− 0.17 to 0.15)

**Table 3:** Effects of Program A (free-choice) and Program B (fixed-sequence) on DASS subscales (adjusted for the stratified factor^a^).

DASS subscales	Program A versus control					Program B versus control				
	Effect (95% CI)	SE	t	p	Estimated effect size (95% CI)^d^	Effect (95% CI)	SE	t	p	Estimated effect size (95% CI)^d^
**Depression**										
3 months^b^	− 0.52 (− 1.48 to 0.45)	0.49	− 1.05	0.29	− 0.08 (− 0.23 to 0.07)	− 0.97 (− 1.94 to − 0.01)	0.49	-1.98	0.048	− 0.16 (− 0.32 to − 0.001)
7 months^b^	0.26 (− 0.79 to 1.32)	0.54	0.49	0.62	0.04 (− 0.12 to 0.19)	0.05 (− 1.00 to 1.10)	0.53	0.10	0.92	0.01 (− 0.15 to 0.16)
Pooled^c^	0.13 (− 0.41 to 0.67)	0.26	0.51	0.62		0.02 (− 0.51 to 0.55)	0.26	0.08	0.94	
**Anxiety**										
3 months^b^	0.08 (− 0.94 to 1.10)	0.52	0.15	0.88	0.01 (− 0.14 to 0.17)	− 0.70 (− 1.72 to 0.32)	0.52	-1.35	0.18	− 0.11 (− 0.26 to 0.05)
7 months^b^	0.25 (− 0.81 to 1.32)	0.54	0.47	0.64	0.04 (− 0.12 to 0.20)	0.06 (− 1.00 to 1.12)	0.54	0.11	0.92	0.01 (− 0.15 to 0.16)
Pooled^c^	0.13 (− 0.41 to 0.66)	0.27	0.46	0.65		0.02 (− 0.52 to 0.56)	0.27	0.07	0.94	

^a^Adjusted for the depression subscale score of DASS 21 (≥ 10 or < 10) in the baseline survey.

^b^Mixed-models for repeated measures ANOVA model analyses were conducted.

^c^Mixed-models for repeated measures conditional growth model analyses were conducted.

^d^Effect sizes were calculated by dividing the estimated effect by a pooled SD at baseline and at follow-ups.

### Subgroup analysis

Among participants with higher depression subscale scores of DASS 21 (≥ 10) at baseline (n = 77 in each of the 3 groups), Program A or B showed no significant intervention effect on either of the two DASS subscales during the follow-up (the pooled effect on depression: t = 0.37, p = 0.71 for Program A; t = 0.70, p = 0.49 for Program B; and the pooled effect on anxiety: t = 0.32, p = 0.75 for Program A; t = − 0.44, p = 0.66 for Program B). The intervention effect of Program A or B was not significant at 3-month follow-up (the effect on depression: t = 0.87, p = 0.39, Cohen’s d = 0.07 [95% CI − 0.26 to 0.40] for Program A; t = − 0.59, p = 0.56, Cohen’s d = − 0.09 [95% CI − 0.41 to 0.24] for Program B; the effect on anxiety: t = 0.87, p = 0.39, Cohen’s d = 0.14 [95% CI − 0.19 to 0.47] for Program A; t = − 0.59, p = 0.56, Cohen’s d = − 0.17 [95% CI − 0.50 to 0.15] for Program B) or at 7-month follow-up (the effect on depression: t = 0.64, p = 0.52, Cohen’s d = 0.17 [95% CI − 0.16 to 0.50] for Program A; t = 0.15, p = 0.88, Cohen’s d = 0.16 [95% CI − 0.16 to 0.49]) for Program B; the effect on anxiety: t = 0.64, p = 0.52, Cohen’s d = 0.08 [95% CI − 0.25 to 0.40] for Program A; t = 0.15, p = 0.88, Cohen’s d = − 0.06 [95% CI − 0.39 to 0.26] for Program B).

### Process evaluation

Table 4 shows the progress of learning in each intervention program. In the Program A group, 264 (83.3%) of participants completed all 6 modules. Completion rates of each module were almost the same (around 84%). In the Program B group, 272 (86.1%) participants completed all 6 modules. Most participants (89.2%) completed module 1 and the proportion of those who completed modules slightly decreased for the later modules (89.2% to 86.7%). Regarding the completion rates, there was no difference between the two intervention groups (p for Fisher’s exact test = 0.33). All participants of the Program A group completed the three CBT-related modules of Program A (i.e., the modules #1-#3); all participants except one of the Program B group completed the five CBT-related modules of Program B (i.e., the modules #2-#6). Table 5 shows the satisfaction and usefulness of the intervention programs at each follow-up period. Regarding the satisfaction at 3-month follow-up survey, there was no difference between two intervention groups (χ^2^(3) = 1.98, p = 0.58). About the usefulness at the 3- and 7-month follow-up surveys, there was also no difference between two intervention groups (χ^2^(3) = 0.98, p = 0.81, and χ^2^(4) = 2.10, p = 0.72, respectively).

**Table 4:** Progress of learning in the Program A (N = 317) and the Program B (N = 316) groups.

Module no.	Contents of program A (free-choice)	The number of completers of modules (%)
1	Behavioral activation	269 (84.9)
2	Cognitive restructuring	269 (84.9)
3	Problem solving	267 (84.2)
4	Assertiveness	266 (83.9)
5	Self-compassion	265 (83.6)
6	Job crafting	264 (83.3)
All six modules		264 (83.3)

Module no.	Contents of program B (fixed-sequence)	The number of completers of modules (%)
1	Transactional model of stress and coping	282 (89.2)
2	Self-case formulation based on cognitive behavioral model	281 (88.9)
3	Behavioral activation	278 (88.0)
4	Cognitive restructuring part 1	276 (87.3)
5	Cognitive restructuring part 2 and Relaxation	274 (86.7)
6	Problem solving	274 (86.7)
All six modules		272 (86.1)

**Table 5:** Satisfaction and usefulness of Program A (free-choice) and Program B (fixed-sequence) at each follow-up period.

	Program A (n = 316)	Program B (n = 316)	*p* value^a^
	N (%)	N (%)	
Satisfaction at 3-month follow-up	Response: 260 (82.0)	Response: 271 (85.8)	0.58
Very satisfied	64 (24.6)	68 (25.1)	
Somewhat satisfied	162 (62.3)	161 (59.4)	
Neither satisfied nor dissatisfied	33 (12.7)	42 (15.5)	
Somewhat dissatisfied	1 (0.4)	0 (0.0)	
Very dissatisfied	0 (0.0)	0 (0.0)	
Usefulness at 3-month follow-up	Response: 259 (81.7)	Response: 272 (86.1)	0.81
Very useful	83 (32.0)	79 (29.0)	
Quite useful	131 (50.6)	138 (50.7)	
Neither useful nor useless	44 (17.0)	54 (19.9)	
Quite useless	1 (0.4)	1 (0.4)	
Very useless	0 (0.0)	0 (0.0)	
Usefulness at 7-month follow-up	Response: 256 (80.8)	Response: 260 (82.3)	0.72
Very useful	111 (43.4)	115 (44.2)	
Quite useful	120 (46.9)	123 (47.3)	
Neither useful nor useless	23 (9.0)	22 (8.5)	
Quite useless	1 (0.4)	0 (0.0)	
Very useless	1 (0.4)	0 (0.0)	

^a^Chi-square test was conducted.

Among 297 (93.4%) of the control group who responded to the question about contamination at 3-month follow-up, 37 (12.5%) answered that they heard about the information related to stress management from those who used the program(s). Among 288 (91.1%) of the control group responded to the question at 7-month follow-up, 90 (31.3%) answered that they did. Regarding the question about adverse events or unintended harm, 250 (78.9%) in the Program A group and 257 (81.3%) in the Program B group answered the question at 7-month follow-up. Out of those, 239 (95.6%) in the Program A group and 253 (98.4%) in the Program B group responded “No.”

## Discussion

The present RCT found that Program B, an iCBT program with a fixed-sequential order of modules, showed a significant intervention effect on improving depressive symptoms at 3-month follow-up among hospital nurses in Vietnam, with a small effect size. Program A, an iCBT program with a free-choice order of modules, did not show a significant intervention effect on any of the primary outcomes. Completion rates were almost the same between these two programs (83.3% in Program A and 86.1% in Program B). There was no significant difference between the two intervention programs in terms of usefulness or satisfaction at the 3-month follow-up. The newly developed smartphone-based iCBT stress management program with fixed sequential order modules may be useful for improving depressive symptoms among nurses in Vietnam.

To our knowledge, the present study is the first to examine whether a smartphone-based multimodule stress management intervention program can improve depressive and anxiety symptoms among nurses in a LMIC. A significant intervention effect was found for the fixed-order program on depressive symptoms at 3-month follow-up. The Cohen’s d effect size (− 0.18) for Program B at 3-month follow-up in this study was similar to or slightly smaller than effect sizes reported from previous universal prevention studies among nurses in the United States^21^ and in the general working populations^18,19^. The small effect size of the program in the present RCT is attributable to lower intensity of the intervention because the iCBT programs in the present study were fully automated and self-guided. A previous systematic review reported that guided iCBT programs with support or feedback from professionals have usually shown a greater effect than non-guided iCBT^44,45^. This may also have led to a small effect size. However, a self-guided program still has merit because of the greater accessibility and lower cost^46^.

Among participants with higher depression score, the intervention effect of Program B on depressive symptoms was not significant at 3- or 7-month follow-up, and the effect size was even smaller than that for the whole sample. This is contrary to previous findings that the effect size to reducing symptoms of stress such as depression was greater for the indicated prevention than the universal prevention in the working population^18^. A possible reason is because the Program B (as well as Program A) was developed as a fully automated, self-guided program. It may be harder for participants already with depressive psychopathology to study CBT from the present program without a professional support or advice. In addition, because the program was developed targeting healthy nurses with minimal symptoms of stress, the program may be perceived by these symptomatic participants as less relevant to their situations. Such characteristics of the program may result in a lower effect on improving depression among those who already had the symptoms. The Program B may be useful when it is applied as the universal prevention rather than as the indicated prevention. Despite the small, limited effect size, the public health impact of the present iCBT program could be scalable in the primary prevention field, when considering its accessibility and minimal cost.

In the present study, Program B showed no significant effect on improving anxiety symptoms at 3- or 7-month follow-up. A previous meta-analysis examining the intervention effect on anxiety and related disorders reported that interventions using exposure strategies had larger effect sizes than those using cognitive and behavioral techniques and cognitive techniques alone^47^. Program B in the present study did not include any exposure technique. This may be among the reasons for the non-significant intervention effect on anxiety symptoms.

The proportions of intervention group participants who completed Programs A and B are similar, suggesting that a free-choice program is not better than a fixed-order program in terms of facilitating the use of this type of smartphone-based stress management program among hospital nurses in Vietnam. This is contradicted previous findings that a free-choice program showed a higher completion rate than a fixed order program in European countries^24^. The superiority of a free-choice program may not be clear in a country with a collectivism- and hierarchy-oriented organizational culture^25^, such as Vietnam. In addition, Program A (free-choice) showed non-significant effects on depression and anxiety symptoms at any follow-up, which were smaller than Program B (fixed-order), which is also contrast to previous finding in Europe^24^. This may be attributable to the difference in the program content rather than the difference in the type of the programs (i.e., free-choice vs fixed-order). Program A contained similar cognitive behavioral techniques (i.e., behavioral activation, cognitive restructuring and problem solving) as Program B, and half of the six modules were overlapped between the two programs. However, there are some differences between the two, which might explain their differential effectiveness on reducing depressive symptoms. With Program B, participants learned the cognitive behavioral model in an early module and then cognitive behavioral skills in later modules. This standard procedure of studying CBT may enhance participants’ understanding the CBT skills and lead to the significant improvement in depressive symptoms compared to the control, while Program A did not use the procedure. Furthermore, two modules of Program B provided the participants with intensive information about the cognitive restructuring skill, which was previously reported to be effective in reducing depression^48^. On the other hand, only one module of Program A was designed to provide this skill. This may make Program A less effective in improving depressive symptoms compared to Program B. It was impossible to make the content of the two programs identical, because the content depended on the type of the program to some extent; it was inappropriate to compare the outcomes between participants who only studied CBT components of Program A and those who studied Program B, because there were no participants who studied CBT components but did not other components of Program A. Further study is needed to compare the effectiveness of free-choice and fixed-order programs on depression and anxiety using strictly comparable free-choice and fixed-order programs in the Asian culture context.

Completion rates were high for both programs. This is attributable to intensive reminders by support staff via online communication tools including instant messages, online group chat, and a hotline telephone service. Satisfaction and usefulness were also high for both programs. This may be attributable to the fact that the programs were developed and culturally modified based on opinions from the target population (i.e., nurses). An iCBT approach may be acceptable and relevant to nurses in Vietnam, if culturally adapted. Not only selecting a case story representing a popular source of stress in the target group, but also selecting wordings and illustrations may be important to increase acceptance and relevance of an iCBT program. A future study is needed to standardize the procedure of cultural adaptation of iCBT programs considering the specific cultures of a country and occupation groups, and other socio-demographic characteristics.

The completion rates, satisfaction, and usefulness of the two programs were similar. The finding is inconsistent with a previous finding reporting a more favorable adherence to a free-choice program than a fixed-order program in European countries^24^. A possible reason is a difference in ways of tailoring between the previous study and the present study. While the previous study used a navigation program to direct users to the most appropriate program based on pre-determined responses concerning their needs and preferences, the present study simply asked users to select one of the free-choice modules. The procedure used here may develop sufficient commitment of users to a selected module for increasing adherence to the program. Another possible reason is cultural difference. In Western countries, selecting options based on one’s decision is a culturally preferred behavior; on the other hand, among Asian countries, the culture is more collectivist and hierarchically oriented, and people tend to feel comfortable to do as others do and to follow instructions^49^. In comparison with participants in Western cultures, participants in the target population might not find being able to select a module from multiple options to be appealing, which might have limited their willingness to commit to the program A. Again, it would be desirable to consider the culture of the target population in designing an iCBT program.

## Limitations

There are several limitations of this study that should be considered. First, participants were recruited from full-time nurses of one big general hospital in Vietnam. Most participants were females, married, and without chronic disease or depressive symptoms at baseline survey. Almost all participants had their own smartphone. The generalization of the present findings to nurses working under different contract conditions and work environments may be limited. Second, all outcomes were measured by self-report, which may be affected by the perceptions of the participants or by situational factors at work. Third, a slight difference in the content between the two intervention programs may also be a limitation in comparing the adherence between the free-choice program and the fixed-order program. The difference of the result between the two programs may be due to other factors than the difference of free-choice or fixed order. Fourth, 12.5% of participants in the control group at 3-month follow-up and 31.3% of participants in the control group at 7-month follow-up answered that they knew information about the intervention programs from their colleagues. This contamination could weaken the intervention effect.
